# Deciphering *O*-glycoprotease substrate preferences with O-Pair Search†

**DOI:** 10.1039/d2mo00244b

**Published:** 2022-12-05

**Authors:** Nicholas M. Riley, Carolyn R. Bertozzi

**Affiliations:** a Department of Chemistry, Sarafan ChEM-H, Stanford University, Stanford, California, USA.; b Howard Hughes Medical Institute, Stanford, California, USA

## Abstract

*O*-Glycoproteases are an emerging class of enzymes that selectively digest glycoproteins at positions decorated with specific *O*-linked glycans. *O*-Glycoprotease substrates range from any *O*-glycoprotein (albeit with specific *O*-glycan modifications) to only glycoproteins harboring specific *O*-glycosylated sequence motifs, such as those found in mucin domains. Their utility for multiple glycoproteomic applications is driving the search to both discover new *O*-glycoproteases and to understand how structural features of characterized *O*-glycoproteases influence their substrate specificities. One challenge of defining *O*-glycoprotease specificity restraints is the need to characterize *O*-glycopeptides with site-specific analysis of *O*-glycosites. Here, we demonstrate how O-Pair Search, a recently developed *O*-glycopeptide-centric identification platform that enables rapid searches and confident *O*-glycosite localization, can be used to determine substrate specificities of various *O*-glycoproteases *de novo* from LC-MS/MS data of *O*-glycopeptides. Using secreted protease of C1 esterase inhibitor (StcE) from enterohemorrhagic *Escherichia coli* and *O*-endoprotease OgpA from *Akkermansia mucinophila*, we explore numerous settings that effect *O*-glycopeptide identification and show how non-specific and semi-tryptic searches of *O*-glycopeptide data can produce candidate cleavage motifs. These putative motifs can be further used to define new protease cleavage settings that lower search times and improve *O*-glycopeptide identifications. We use this platform to generate a consensus motif for the recently characterized immunomodulating metalloprotease (IMPa) from *Pseudomonas aeruginosa* and show that IMPa is a favorable *O*-glycoprotease for characterizing densely *O*-glycosylated mucin-domain glycoproteins.

## 1. Introduction

Glycosylation is a fundamental attribute of the extracellular proteome, but characterizing glycoproteins remains challenging.^1–3^ Dedicated efforts to improve mass spectrometry (MS)-based glycoproteomics methodology have significantly increased our ability to analyze intact glycopeptides, which can provide site-specific characterization of glycoproteins to capture macro- and microheterogeneity across the glycoproteome.^4–9^ Thanks, in part, to the presence of a consensus *N*-glycosylation sequence motif, location of *N*-glycosites in regions accessible by canonical proteases, effective endoglycosidases (*i.e*., PNGaseF), and favorable gas-phase fragmentation behavior in ubiquitous tandem MS approaches that use collision-based dissociation, thousands of *N*-glycopeptides and *N*-glycosites can now be profiled in a single experiment.^10–16^ Conversely, *O*-glycoproteins enjoy none of these analytical benefits, making *O*-glycosite characterization a significantly more challenging task that requires new and innovative tools.^17–22^

An exciting development in the glycoproteomics field has been the emergence of *O*-glycoproteases, which are endoproteases requiring a combination of glycan and amino acid sequence characteristics to cleave the peptide backbone of *O*-glycoproteins.^23^ Specific examples include: secreted protease of C1 esterase inhibitor (StcE) from enterohemorrhagic *Escherichia coli*;^24–26^ *O*-endoprotease OgpA (commercially available as OpeRATOR) and M60-like proteases AM0627, AM0908, and AM1514 from *Akkermansia mucinophila*;^27–33^ zinc-metalloendopeptidase CpaA from several *Acinetobacter* strains;^34^ BT4244 from *Bacteroides thetaiotaomicron*;^31,32,35^ zinc metalloproteinase C (ZmpC) from *Streptococcus pneumoniae*;^31,36^ SmEnhancin from *Serratia marcescens*;^37,38^ and immunomodulating metalloprotease (IMPa) from *Pseudomonas aeruginosa*.^35,39,40^ These enzymes have been adapted as a means to selectively deplete specific classes of *O*-glycoproteins (*e.g*., mucin-domain glycoproteins) from live cell populations, in addition to being used in catalytically inactive forms for imaging and enrichment purposes.^30,31,41–43^ Perhaps the most immediate utility for *O*-glycoproteases, however, is their use in glycoproteomic workflows to generate *O*-glycopeptides that are more amenable to sequencing by tandem MS for site-specific characterization of previously intractable *O*-glycoproteins.^20^ Regardless of their application, understanding substrate preference(s) is fundamental to defining the mechanism of action and biological role of various *O*-glycoproteases.^23^ The unique combinations of peptide sequence and *O*-glycoforms that govern *O*-glycoprotease activity mean that substrate preferences must be assessed with *O*-glycoproteomics of the (glyco)peptide cleavage products.

Current efforts to define *O*-glycoprotease substrate preferences are low-throughput, requiring manual *de novo* sequencing of peptides, *O*-glycosite localization, and sequence alignment. *O*-Glycosite localization is especially important to accurately describe how both proximity of glycosites to proteolytic cleavage sites and also presence of certain glycan types contribute to recognition and cleavage specificities of individual *O*-glycoproteases. Here we explore how O-Pair Search, a recently developed glycoproteomics search engine specifically designed for *O*-glycopeptides,^44^ can expedite this process. O-Pair Search offers several key advantages that directly benefit *O*-glycoprotease substance elucidation, including (1) rapid search times that enable larger glycan databases and consideration of multiple *O*-glycosites per peptide, (2) localization capabilities for multiple *O*-glycosites in a single peptide with confidence scores based on localization probabilities, and (3) identification quality categorization that permits straightforward filtering of identifications with localized *O*-glycosites. Furthermore, MetaMorpheus, the free and open-source environment that houses O-Pair Search, allows for user-defined protease settings that can be created for *O*-glycoproteases based on cleavage preferences gleaned from semi-tryptic and non-specific searches.^45,46^ We demonstrate how to use O-Pair Search to decipher *O*-glycoprotease substrate preferences using the well-defined examples of StcE and OgpA, and we show how defining the cleavage patterns in MetaMorpheus can improve *O*-glycopeptide identifications. We then use our approach to explore how the presence of sialic acids affect the cleavage specificities of OgpA, generate a consensus cleavage motif for IMPa, and show the benefits IMPa can offer for *O*-glycopeptide identification over StcE and OgpA. Ultimately, this work highlights the flexibility and data quality offered by O-Pair Search to aid future efforts to characterize the growing list of *O*-glycoproteases.

## Experimental

### Proteolytic digestion

Recombinantly expressed MUC16, CD43, GP1bα (CD42b), podocalyxin, and PSGL-1, were purchased from R&D Systems (5609-MU, 9680-CD, 4067-gP, 1658-PD, and 3345-PS, respectively). StcE was expressed and purified as previously described.^26^ Briefly, *E. coli* BL21(DE3) was transformed with pET28b-StcE-Δ35-NHis and grown at 37 °C until an optical density of 0.6–0.8 was reached. The culture was then induced with 0.3 mM IPTG and incubated at 20 °C overnight. Cells were lysed in 20 mM HEPES pH 7.5, 500 mM NaCl using a probe tip sonicator. Lysates were applied to HisTrap HP columns (GE Healthcare Life Sciences) using a GE ÄKTA Pure FPLC. After washing with 20 column volumes of lysis buffer + 20 mM imidazole, elution was performed using a 15 min linear gradient from 20 mM imidazole to 250 mM imidazole. Pooled fractions for each enzyme were concentrated using Amicon Ultra 30 kDa MWCO filters (Millipore Sigma), then snap frozen in liquid nitrogen and stored at −80 °C. OgpA and a pan sialidase were purchased from Genovis under the names OpeRATOR and SialEXO (G1-OP1–020 and G1-SM1–020, respectively). IMPa was purchased from New England BioLabs as *O*-glycoprotease (P0761S). For each condition, 5 mg of each recombinant protein was digested, all digestions were conducted in 100 mM ammonium bicarbonate, and all digestions occurred during a three-hour incubation at 37 °C. StcE and IMPa digestions were conducted at a 1 : 10 protease : protein ratio by weight. OgpA digests were conducted at a 1 : 1 protease units : protein weight ratio, as directed by the manufacturer. When sialidase was added (for OgpA and IMPa digestions as described in the text), SialEXO was co-incubated with the *O*-glycoprotease. Following *O*-glycoprotease digestion, 1 μl PNGaseF (New England Biolabs, P0709S, diluted to 10 000 U ml^−1^ in PBS) was added to each digestion for an overnight (~12 hour) incubation at 37 °C. TCEP and CAA (Sigma Aldrich) were then added to final concentrations of 10 mM and 40 mM, respectively, followed by sequencing grade trypsin (Promega) at a 1 : 25 protease : protein ratio by weight. Samples were incubated for 12 hours at room temperature. Reactions were quenched by dilution with 500 μl of 0.2% formic acid (FA) in water and peptides were desalted using 10 mg/1 ml Strata-X columns (Phenomenex). Briefly, columns were wet with 1 ml of acetonitrile followed by 1 ml of 0.2% FA. Acidified peptides were loaded onto the columns and washed with 300 μl of 0.2% FA. Peptides were eluted with 400 μl of 0.2% FA, 80% acetonitrile, dried *via* lyophilization, then resuspended in 10 μl of 0.2% FA. All data reported here are results from two technical replicates of these conditions, *i.e*., each proteolytic digestion on each glycoprotein was performed twice in tandem and data were collected and analyzed separately for each replicate.

### LC-MS/MS

Data was acquired using product-dependent triggering of EThcD scans (*i.e*., an electron transfer dissociation with supplemental beam-type collisional activation) as previously described.^33,47^ Approximately 2 μg of peptides were injected on the column for each sample (one protein digest per run). Peptides were separated over a 25 cm Aurora Series UHPLC reversed phase LC emitter column (75 μm inner diameter packed with 1.6 μm, 160 Å, C18 particles, IonOpticks) that was heated to 40 °C by a Sonation PRSO-V2 column heater. A Dionex Ultimate 3000 RPLC nano system (Thermo Fisher Scientific) with an integrated loading pump was used for online liquid chromatography using mobile phases A (0.2% FA in water) and B (0.2% FA in acetonitrile). Peptides were loaded onto a trap column (Acclaim PepMap 100 C18, 5 μm particles, 20 mm length, Thermo Fisher Scientific) at 5 μl min^−1^, which was put in line with the analytical column 5.5 min into the acquisition. Gradient elution was performed at 300 nl min^−1^. The gradient was held at 0% B for the first 6 min of the analysis, followed by an increase from 0% to 5% B from 6 to 6.5 min, an increase from 5% to 22% B from 6.5 to 156.5 min, an increase from 22% to 90% B from 156.5 to 160 min, isocratic flow at 90% B from 160 to 164 min, and a re-equilibration at 0% for 16 min for a total analysis time of 180 min. Eluted peptides were analyzed on an Orbitrap Fusion Tribrid MS system (Thermo Fisher Scientific). Precursors were ionized using an nanospray flex ionization source (Thermo Fisher Scientific) held at +2.2 kV compared to ground, and the inlet capillary temperature was held at 275 °C. Survey scans of peptide precursors were collected in the Orbitrap from *m*/*z* 400 to 1800 with a normalized AGC target of 100% (400 000 charges), a maximum injection time of 50 ms, and a resolution of 60 000 at *m*/*z* 200. Monoisotopic precursor selection was enabled for peptide isotopic distributions, precursors of *z* = 2 to 8 were selected for data-dependent MS/MS scans for 3 s of cycle time, and dynamic exclusion was enabled with a repeat count of 2, repeat duration of 20 s, and exclusion duration of 20 s. Priority filters were set to favor highest precursor charge states and lowest precursor m/z values. An isolation window of 2 *m*/*z* was used to select precursor ions with the quadrupole. EThcD scans were collected in product-dependent fashion,^48–50^ where the presence of oxonium ions (*m*/*z* 126.055, 138.0549, 144.0655, 168.0654, 186.076, 204.0865, 274.0921, 292.1027, and 366.1395) in a “scouting” higher-energy collisional dissociation (HCD) MS/MS scan triggered acquisition of a second MS/MS scan. The “scout HCD” scan used an automated scan range determination and a first mass of 100 Th, a normalized collision energy of 36, a normalized AGC target value of 100% (50 000 charges), a maximum injection time setting of Auto (54 ms), and a resolution of 30 000 at *m*/*z* 200. If at least four of the nine listed oxonium ions were present in the scout HCD scan within a ±15 ppm tolerance and were among the 20 most intense peaks, an EThcD MS/MS scan was triggered that used calibrated charge dependent parameters for calculating reagent AGC targets and ion–ion reaction times,^51^ a supplemental collision energy of 25, a scan range of *m*/*z* 200 to 4000, a maximum injection time of 400 ms, a normalized AGC target of 200% (100 000 charges), and a resolution of 60 000 at *m*/*z* 200.

### Data analysis

**Fig. 1:**
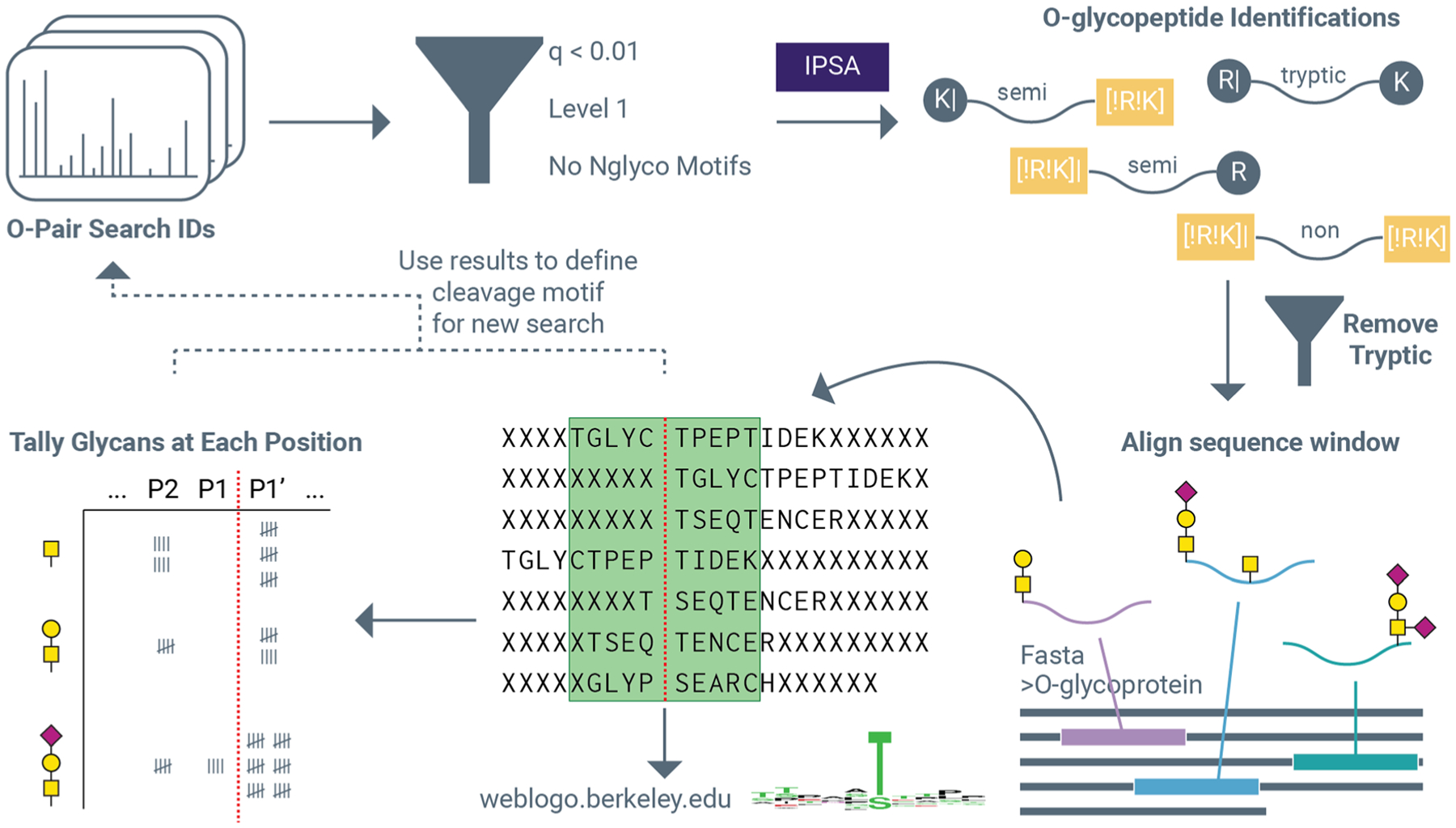
Strategy to decipher *O*-glycoprotease substrates with O-Pair Search results. O-Pair Search identifications are filtered to retain high confidence *O*-glycopeptide identifications, checked with the Interactive Peptide Spectral Annotator (IPSA), and filtered to remove any fully tryptic peptides that would confound cleavage motif analysis. *O*-Glycopeptide sequences are then mapped on their protein sequence using the FASTA file used in the search and aligned in a ±5 residue window around the cleavage point. Semi-tryptic peptides were aligned based on their non-tryptic N- or C-terminus, and fully non-tryptic peptides were aligned using both termini using two separate sequence window entries. Ten residue sequence windows were then input to https://weblogo.berkeley.edu to generate minimum sequence motifs, and *O*-glycosites and their occupying *O*-glycans were tallied for each position. This information can then be used to define new cleavage parameters for use in a new O-Pair Search analysis.

All raw data were searched using O-Pair Search implemented in MetaMorpheus (0.0.320), which is available at https://github.com/smith-chem-wisc/MetaMorpheus.^44^ All searches were performed on a PC running Windows 10 Education, with two2.20 GHz Intel Xeon Silver 4114 CPU processors with 64 Gb of installed RAM. Sixteen cores were used per search. Files for digestions of each protein from a given digestion condition (*e.g*., all five proteins digested with StcE and trypsin) were searched together in batches with a FASTA file containing Uniprot^52^-derived sequences from all five proteins as described by their sequences from the vendor. O-Pair Search is capable of searching larger protein databases with reasonable search times, as discussed in the original description of the O-Pair Search algorithm.^44^ Here, we elected to keep the database to known glycoprotein standards in the samples to help minimize any false hits, as has been suggested elsewhere.^53^ Multiple parameters were tested as indicated in the text, which are further explained in Table S1 (ESI†). The standard search parameters are also described in Table S1 (ESI†) and went as follows. The “Glyco Search” option was selected, where the *O*-glycopeptide search feature was enabled with an *O*-glycan database of 22 glycans (Data 1, ESI†). For glycans in the database, N denotes HexNAc, H denotes Hex, A denotes NeuAc, G denotes NeuGc, and F denotes Fucose The “Keep top N candidates” feature was set to 50, and Data Type was set as HCD with Child Scan Dissociation set as EThcD. The “Maximum OGlycan Allowed” setting was set to 4, where this number represents both the maximum number of *O*-glycan modifications that could occur on a glycopeptide candidate and the number of times each *O*-glycan could occur per peptide. Under Search Parameters, both “Use Provided Precursor” and “Deconvolute Precursors” were checked. Peak trimming was not enabled. *In silico* digestion parameters were set to generate decoy proteins using reversed sequences, and the initiator methionine feature was set to “Variable”. The maximum modification isoforms allowed was 1024, and nonspecific digestion was enabled for peptides ranging from 5 to 60 residues. Precursor and product mass tolerances were 10 and 20 ppm, respectively, and the minimum score allowed was 3. Modifications were set as carbamidomethyl on C as fixed, and oxidation on M and deamidation on N as variable. Deviations from these settings explored in this study are described in Table S2 (ESI†). The oglyco.psmtsv results file was used for further data processing. Non-modified, non-fully tryptic peptides accounted for fewer than 2% of identifications in all searches, so they were omitted from further analyses. Note, O-Pair Search returns a single identification representing two spectra, both a beam-type collision-induced dissociation (beamCID, referred to as higher-energy collisional dissociation [HCD] on some instrument platforms) and an electron transfer dissociation with supplemental beam-type collisional activation (EThcD) spectrum. Identifications are made using the beamCID spectrum, and the associated EThcD spectrum is used to localize *O*-glycosites. Identifications were filtered to include only target matches (T) and identifications with a *q*-value <0.01. *O*-Glycopeptide identifications were further filtered to include only Level 1 identifications, which include only identifications with confident and unambiguous *O*-glycosite localization (localization probability >0.75), and to exclude *O*-glycopeptides that contained an *N*-glycosylation sequon (N–X–S/T). Although it is possible to have *O*-glycans present on S or T residues within the *N*-sequon, this remains a confounding variable in both *N*- and *O*-glycoproteomics experiments. There remains a lack of consensus across the field of how often this happens, as it is an informatically challenging problem that is insufficiently handled by most software available. That said, it is thought that the rate of *O*-glycosylation at an *N*-sequon is substantially lower than *N*-glycosylation at that position. It is for these reasons that we chose to treat peptides with PNGaseF and omit any *O*-glycopeptides that had an *N*-sequon in this study. Identified spectra were manually inspected using the Interactive Peptide Spectral Annotator (https://www.interactivepeptidespectralannotator.com/PeptideAnnotator.html).^54^ Fully tryptic peptides were removed from consideration prior to *O*-glycoprotease substrate cleavage determination. Peptides were then categorized as “Nterm” if they had non-tryptic cleavage at their N-terminus, “Cterm” if they had non-tryptic cleavage at their C-terminus, or “Both” if they were fully non-tryptic. Sequence windows for motif generation were obtained by mapping filtered *O*-glycopeptide identifications onto FASTA sequences using the N-terminal residue of the *O*-glycopeptide as the P1′ alignment point for “N-term” identifications or the C-terminal residue as the P1 alignment point for “C-term” identifications. For peptides categorized as “Both”, two sequence windows were created, one each for N- and C-terminal alignment at P1′ and P1, respectively. For each sequence window, five residues upstream (P5 → P1) and five residues downstream (P1′ → P5′) were extracted the alignment (*i.e*., cleavage) point, and glycosites and their occupying *O*-glycans were tallied at each sequence position. Ten residue sequence windows (P5 → P5′) were input into https://weblogo.berkeley.edu to generate minimum sequence motifs.^55^ Fig. 1 summarizes this process graphically. Search times reported were taken directly from O-Pair Search output files. The percent of *O*-glycosylated serine and threonine residues was determined by counting the number of glycosylated residues at a given position relative to the total number of serine and threonine residues at that position for identified sequences. Serine and threonine counts were summed, so this is an aggregate value for both residues. For example, if an entire dataset contained only 10 species, all with the sequence SPEPTIDE, with 9 of them reporting an *O*-glycan at the first S (*i.e*., position P1′), the percentage of glycosylated S at P1′ would be reported as 90%. This counting process was done for S and T residues at positions P5 → P5′ for all identifications, and percentages of modified residues were calculated for each position individually. All data reported is the average of two replicates unless otherwise reported. Byonic searches done for brief comparisons described in the text used a total common max value set to 2 and a total rare max value set to 2. The 22 *O*-glycan database was used, and each *O*-glycan was set as common2. Other modifications were: carbamidomethyl at cysteine (+57.021644, fixed), oxidation at methionine (+15.994915, rare2), and deamidation at asparagine (+0.984016, rare1). Cleavage specificity was set as semi-specific for C-terminal to R and K residues (semi tryptic) with three missed cleavages allowed. Precursor mass tolerance was set to 10 ppm with fragment mass tolerance(s) set to 20 ppm with fragmentation set to HCD & EThcD and protein FDR set to 1%. Filtering Byonic search results is necessary to retain only high-quality identifications and minimize false positives. Filtering metrics included a Byonic score greater than or equal to 200, a logProb value greater than or equal to 2, a deltaMod score greater than 10, and peptide length greater than 4 residues. Mass spectrometry raw data, a FASTA sequence database, and O-Pair Search results have been deposited to the ProteomeXchange Consortium *via* the PRIDE partner repository with the dataset identifier PXD035775.^56^

## Results and discussion

Mapping the substrate preferences of *O*-glycoproteases requires the ability to unambiguously sequence *O*-glycopeptides with confident *O*-glycosite localization. Generally, this is accomplished using LC-MS/MS-based glycoproteomics. The need for *O*-glycosite localization means electron-based tandem MS fragmentation is typically required because *O*-glycan modifications are labile and not retained on peptide-backbone fragments under most collision-based tandem MS dissociation conditions.^47,57^ This remains true even for *O*-glycoproteases, *e.g*., OgpA, that generate *O*-glycosites at the N-terminus of peptides by default of their cleavage activity, because internal *O*-glycosites present in the peptide sequence can lead to false determination of the glycan composition at the N-terminus under collisional dissociation.^58^ To maximize our data quality and ability to localize *O*-glycosites, we used a product-dependent triggering method, where beam type-collisional dissociation MS/MS spectra are collected for precursor ions in data-dependent fashion. Every spectrum that contains glycan-specific oxonium ions then triggers collection of an associated EThcD MS/MS spectrum for the same precursor ion, creating paired beamCID-EThcD spectra for each potential glycopeptide precursor ion. Several glycoproteomics software platforms are equipped to identify *O*-glycopeptides from both spectrum types,^59–63^ but O-Pair Search is a tool we developed to specifically handle this type of *O*-glycoproteomics data.^44^ O-Pair Search uses the paired spectra in concert to identify *O*-glycopeptides and localize *O*-glycosites even for peptides with multiple *O*-glycosites, and it provides rapid searches through a fragment ion index approach to enable reasonable search times even with medium sized (~20–50) glycan composition databases and with semi-tryptic and non-specific protease settings that considerably expand search space.

With these strengths in mind, we sought to explore how to leverage the advantages of O-Pair Search to decipher the complex substrate preferences of several *O*-glycoproteases. Fig. 1 describes our general approach that starts with identifications from O-Pair Search. First, filtering for a 1% false discovery rate at the *O*-glycopeptide level (which does not include non-modified sequences) and for Level 1 identifications that have all potential *O*-glycosites localized ensures only high-quality *O*-glycopeptide identifications are retained. *O*-Glycopeptides that contained an *N*-glycosylation sequon (N–X–S/T, where X is any amino acid except proline) were also removed to minimize potential confounding issues from incomplete *N*-glycan removal, and correct assignments were verified for randomly selected identifications using the Interactive Peptide Spectral Annotator (IPSA).^54^ The number of *O*-glycopeptides spectral matches (*O*-glycoPSMs) and related unique *O*-glycopeptides and *O*-glycosites reported throughout this study are all derived from data following these filtering steps. To generate cleavage motifs specific to the *O*-glycoprotease in question, fully tryptic *O*-glycopeptide identifications were filtered out prior to aligning *O*-glycopeptide sequences to their correct positions (based on their non-tryptic termini, as described in the methods) within the protein FASTA sequence, and a 10-residue sequence window (±5 residues from the cleavage site) was used to generate the peptide sequence motif component of the minimum *O*-glycoprotease cleavage motif using https://weblogo.berkeley.edu. This strategy can be used to quickly generate motifs with non-specific and semi-tryptic search settings when nothing is known about cleavage preferences, which can then be used to define new cleavage parameters for a refined O-Pair Search analysis.

**Fig. 2:**
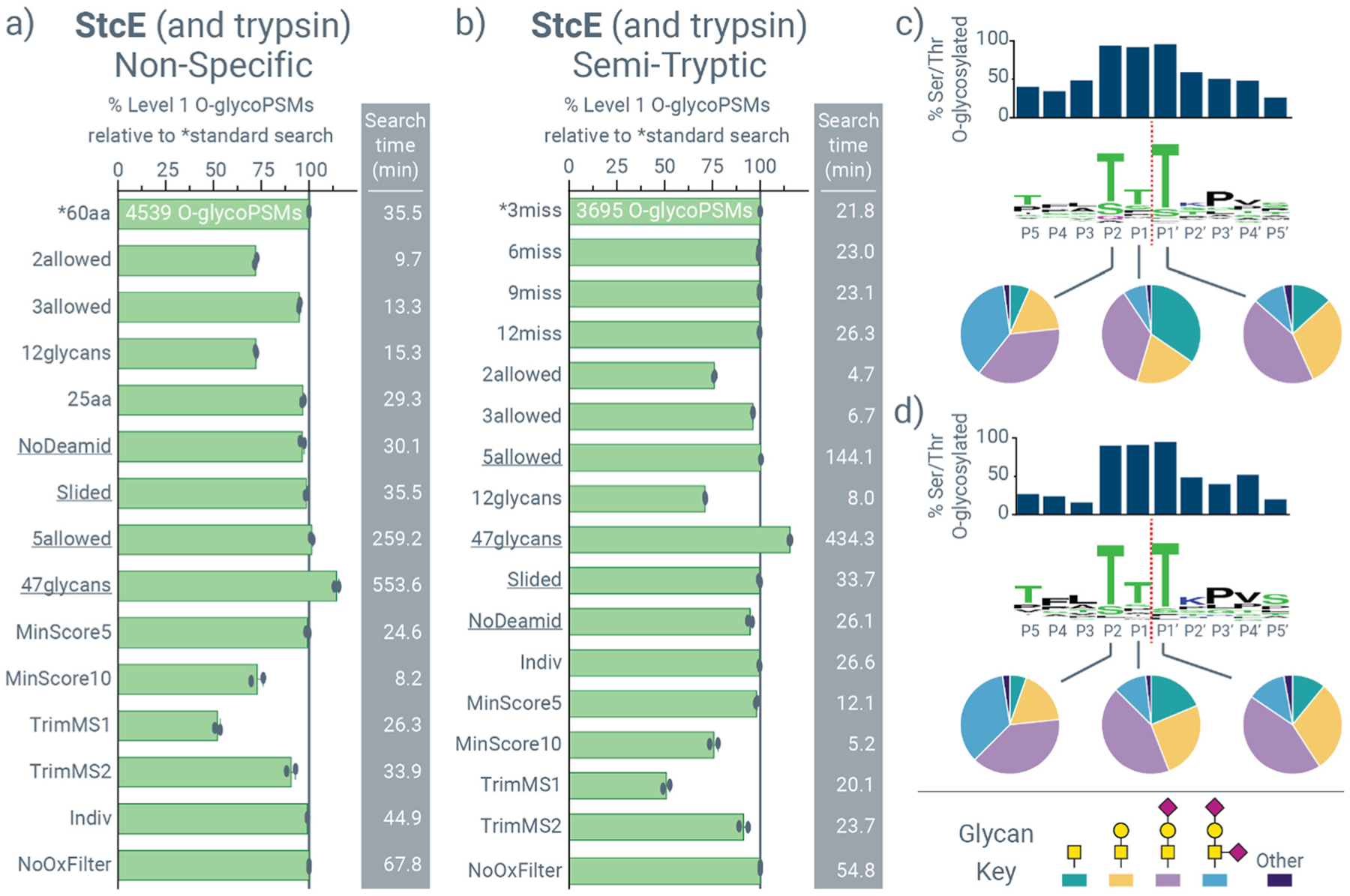
Exploring O-Pair Search settings for identifying *O*-glycopeptides generated from sequential StcE and trypsin digestion. *O*-GlycoPSM identifications for (a) non-specific searches and (b) semi-tryptic searches of mucin-domain *O*-glycoproteins digested sequentially with StcE and trypsin (StcE + trypsin). All identifications are scaled to the standard search settings (*, the top bar in each graph), and total number of identifications are provided for standard searches. Average search times in minutes are provided to the right of each bar graph, bars represent the average of two replicates that are also provided as separate data points, and search settings are explained further in Tables S1 and S2 (ESI†). Peptide-glycan cleavage motifs are shown for StcE cleavage generated by (c) the standard non-specific search and (d) the standard semi-tryptic search. Sequence motifs in the middle indicates amino acid specificities at each position, with cleavage between P1 and P1′ residues (red dotted line). Bar graphs above the sequence motifs show the percent of serine and threonine residues observed at a given location that were *O*-glycosylated, and pie graphs show the distribution of glycans observed at P2, P1, and P1′.

Fig. 2 highlights this motif discovery process for data obtained from a sequential StcE-trypsin digestion of a panel of five *O*-glycoprotein standards with *O*-glycosites harboring mainly core-1 and core-2 *O*-glycans with minor contributions from other mucin-type *O*-glycans. These standards have *O*-glycosites within and outside of mucin domains, providing *O*-glycosite-rich substrates to investigate *O*-glycoprotease cleavage. Fig. 2a and b show the number of *O*-glycoPSM identifications obtained from non-specific and semi-tryptic search, respectively, using a variety of user-defined search parameters (italicized in the text for emphasis). Table S1 (ESI†) provides descriptions of these parameters and their values for a “standard” search that serves as a benchmark in this study, and Table S2 (ESI†) describes the changes to each of the parameters shown. Identifications are scaled to the standard search, whose number of *O*-glycoPSMs are provided, and search times for all searches are provided to the right of the bar graph. Most parameters were chosen in an attempt to explore reduced search times, but the four underlined searches (*NoDeamid*, *Slided*, *5allowed*, and *47glycans*) were chosen to explore a potential increase in the number of identifications. Note, search times are highly dependent on hardware used. Data throughout this manuscript are representative of using 16 cores per search on a system described in the Methods. Search times on different hardware configurations will likely vary, but we designed these search conditions to match hardware that may be available in a typical (glyco)proteomics laboratory.

Semi-tryptic searches will not consider *O*-glycopeptides generated from *O*-glycoprotease cleavage at both termini, while non-specific searches will; this difference likely accounts for the difference in identifications between the two. Interestingly, for both non-specific and semi-tryptic searches of data from StcE digests, few parameters increased identifications, except for including a larger glycan database (47 instead of 22 glycan compositions, available in Data 1, ESI†), which includes more extended core-1 and core-2 structures. Conversely, using a smaller O-glycan composition database decreased identifications by approximately one fourth (*vide infra*), underscoring the importance of using appropriate glycan databases in glycoproteomic searches.^53^ Decreasing the number of glycosites considered per peptide from the four *O*-glycosites used in the standard search substantially lowered search times, but the number of *O*-glycoPSMs were negatively affected, especially when only considering two *O*-glycosites per peptide. This has implications for search *O*-glycopeptide data from *O*-glycoprotease cleavage with other search engines that only consider one glycosite per peptide, or that do not have the speed to effectively manage searches that consider >2 *O*-glycosites without a significantly truncated *O*-glycan database.

Straightforward comparisons of most of these parameters to searches with the popular glycoproteomics software Byonic are not possible because the combinatorial space of multiple *O*-glycans on several potential *O*-glycosites causes prohibitively long Byonic searches. Indeed, this was one of the original motivations of developing O-Pair Search and is explored in prior work.^44^ For example, a *non-specific search* of StcE-trypsin data with Byonic with a simple *three O-glycan database* and only *3 glycans allowed per peptide* required 2380 minutes (*i.e*., 1 day, 15 hours, and 40 minutes) for a single raw file, compared to 13.3 minutes for five raw files with O-Pair Search using a 22-*glycan database* with “*3allowed*”. To enable a reasonable comparison with Byonic, we performed a semi-tryptic search of StcE-trypsin data using Byonic with *3 missed cleavages* allowed (the same as standard settings for O-Pair Search) and the 22-*glycan database*, but with only 2 *O*-glycans allowed (*i.e*., a *common2* setting in Byonic). This corresponds to the “*2allowed*” data in Fig. 2b discussed above. Byonic semi-tryptic searches allowing only two *O*-glycans per sequence required 1951 and 2043 minutes (~33 hours on average) for replicates 1 and 2, respectively. This is ~425-fold longer than the average search time (4.7 minutes) for a semi-tryptic search with *3 missed cleavages*, a 22-*glycan database*, and a simplified “*2allowed*” setting in O-Pair Search. Byonic identified ~73% of the total *O*-glycopeptide spectra identified by the “*2allowed*” O-Pair Search (2048 *vs*. 2804 average *O*-glycoPSMs), which is only ~55% of the standard O-Pair Search that uses a “*4allowed*” setting (Fig. S1, ESI†). O-Pair Search identified all but 2 of the 186 *O*-glycosites identified by Byonic. When attempting to search for more *O*-glycans per peptide with Byonic (*i.e*., a *common3* setting in Byonic), even with a smaller *O*-glycan database, searches took over 5 days per raw file and were cancelled before they could finish. These data demonstrate the incompatibly of a conventional software tool like Byonic for exploring the parameters described in this work.

Searching files from each digest individually rather than in a single batch for a given digestion condition resulted in the same number of identifications, but actually took ~25% longer to search, not including time to aggregate results from each after searches were done. Similarly, not using an oxonium ion filter as a requirement to consider spectra for *O*-glycopeptide identification did not affect identifications, but it did result in almost double the amount of search time because more spectra had to be considered, effectively demonstrating the benefits of oxonium ion filters that have also been explored in other software platforms.^60,64^ Requiring a *minimum Morpheus score*^65^ of five had practically no effect on identifications, although a requirement of a score of ten did reduce identifications, showing that most beamCID spectra have at least five and often more than ten product ions matched that contribute to identifications. Trimming MS1 peaks using a relative intensity threshold had more of a negative effect on identifications than trimming MS2 peaks, indicating that most matched product ions in MS2 spectra are relatively abundant while successfully-sequenced *O*-glycopeptide precursor ions are not necessarily the most abundant species in MS1 spectra when they are selected for MS/MS. Perhaps surprisingly, a peptide length of 25 residues *vs*. the standard 60 residues did not have a dramatic change in identifications for non-specific searches, nor did increasing the number of missed cleavages from three to six, nine, or twelve in semi-tryptic searches.

The standard parameters for non-specific and semi-tryptic searches were then used to generate cleavage motifs for StcE, as shown in Fig. 2c and d, respectively. These motifs have three components, with the typical peptide sequence motif in the middle. Above the sequence motif is a bar graph that shows the percentage of serine and threonine residues at a given position that were detected as *O*-glycosylated. Below the sequence motif are pie graphs that show the distribution of glycans observed at indicated positions. These three data combine to describe the cleavage motif for StcE, which look remarkably similar to the previous cleavage motif generated by manual analysis^26^ and also to each other. We observe from these data that StcE’s cleavage motif requires a T/S–X–T/S sequence at the P2–P1–P1′ positions, where X can be any amino acid, but is often a threonine or serine residue as well. Based on these data, StcE also permits a broad range of *O*-glycosylation at each of these positions, including sialylated and non-sialylated core-1 and core-2 *O*-glycans, with the threonine or serine residues at P2 and P1′ to be *O*-glycosylated at effectively 100% frequency. One nuanced feature about StcE that was originally reported for its cleavage motif that is less easy to discern from our strategy is the requirement of *O*-glycosylation at P2 without a requirement at P1′.^26^ However, our strategy permits rapid determination of a putative cleavage motif that can be tested on synthesized standards with defined features, as is typically required for nuanced features.

**Fig. 3:**
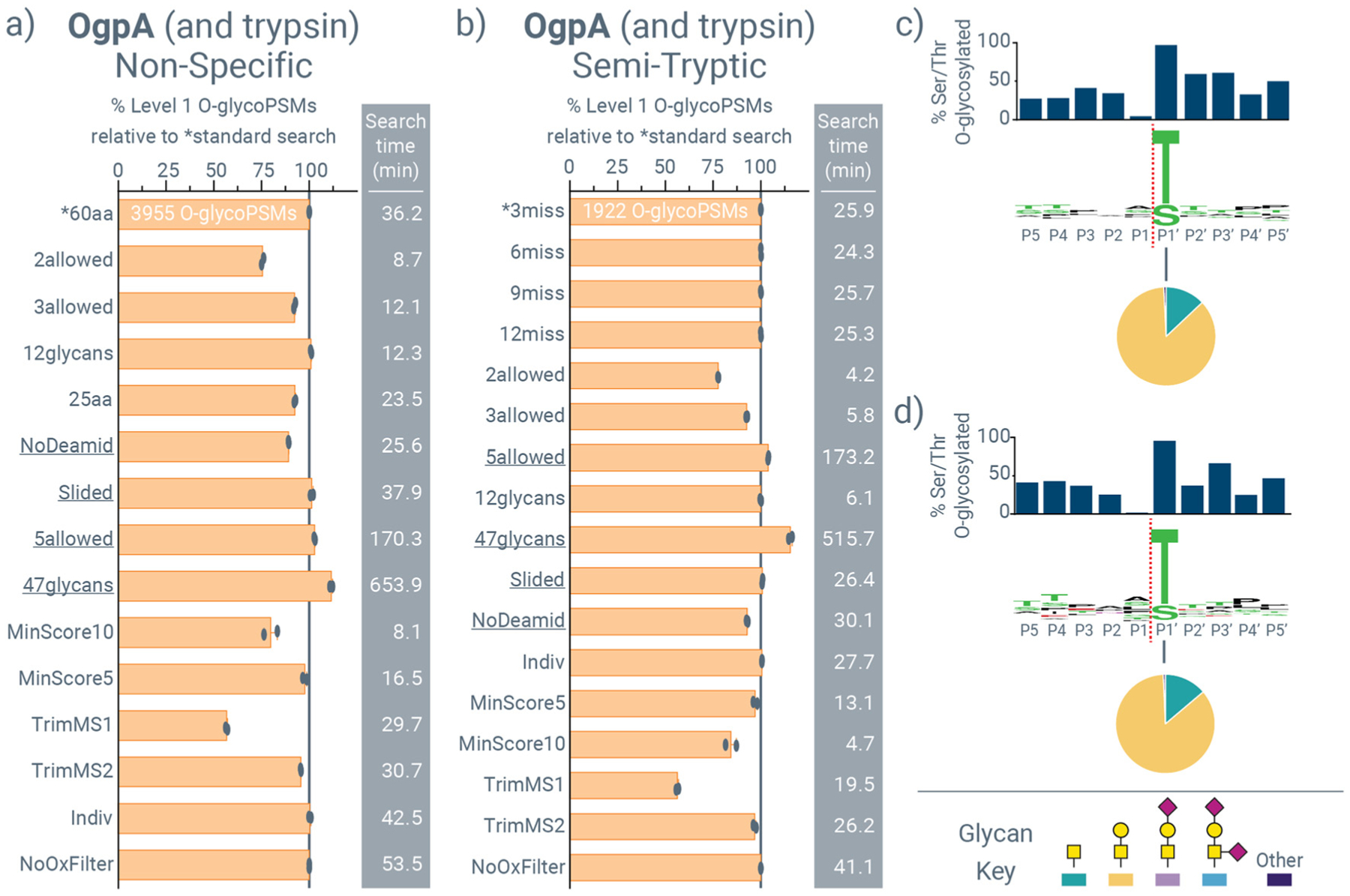
Exploring O-Pair Search settings for identifying *O*-glycopeptides generated from sequential OgpA and trypsin digestion. *O*-GlycoPSM identifications for (a) non-specific searches and (b) semi-tryptic searches of mucin-domain *O*-glycoproteins digested sequentially with OgpA and trypsin (OgpA + trypsin). OgpA digestion here is done concurrently with sialidase treatment according to standard practice. All identifications are scaled to the standard search settings (*, the top bar in each graph), and total number of identifications are provided for standard searches. Average search times in minutes are provided to the right of each bar graph, bars represent the average of two replicates that are also provided as separate data points, and search settings are explained further in Tables S1 and S2 (ESI†). Peptide-glycan cleavage motifs are shown for OgpA cleavage generated by (c) the standard non-specific search and (d) the standard semi-tryptic search. Sequence motifs in the middle indicates amino acid specificities at each position, with cleavage between P1 and P1′ residues (red dotted line). Bar graphs above the sequence motifs show the percent of serine and threonine residues observed at a given location that were *O*-glycosylated, and pie graphs show the distribution of glycans observed at P1′.

Fig. 3 provides similar data using OgpA as a glycoprotease with co-incubation with a sialidase (as recommended by the manufacturer) and subsequent trypsin digestion. Many trends in identifications that arise from changing search parameters are the same between StcE and OgpA data. For both *O*-glycoproteases, semi-tryptic searches were slightly faster than non-specific searches, as expected. The most substantial increase in *O*-glycoPSMs for OgpA data was also with the 47-*glycan database*, and the presence of multiple *O*-glycosites in *O*-glycopeptides in OgpA proteolysis, shown by decreases in identifications when considering two or three instead of four *O*-glycosites per peptide, matches previous reports.^58^ Interestingly, five *O*-glycosites per peptide did not substantially increase identifications for StcE data (but did increase search times greater than seven-fold). Conversely, this parameter did provide a slight increase for OgpA data. This result likely indicates a combination of factors, including a potentially decreased cleavage efficiency in OgpA relative to StcE and a heavier dependence on glycan type for cleavage to occur with OgpA (which also matches previous reports^66^). Another interesting difference in StcE and OgpA data is the effect of the 12-*glycan versus* 22-*glycan database* (Data 1, ESI†). The 22-*glycan database* extends the 12-*glycan database* (which is a common *O*-glycan database in *O*-glycoproteomic applications^67^) to include the same glycans but with NeuAc and NeuGc sialic acids, whereas the 12-*glycan database* only has NeuAc sialic acids. NeuGc sialic acids are not found in typical human glycoproteins,^68^ but are in the recombinant proteins used here generated from CHO and NS0 cells. In StcE digests (which did not include sialidase treatment), the inclusion of the NeuGc-containing *O*-glycans in the 22-*glycan database* resulted in more identifications than the 12-*glycan database* (Fig. 2a and b). OgpA, however, is reported be to be less efficient at cleaving in the presence of sialylated *O*-glycans,^27,28^ prompting the co-incubation with a sialidase as noted above. This effectively negates the difference in identifications between the 12- and 22-*glycan databases* for OgpA data. Counter to this, the 47-*glycan database* adds other glycan compositions that do not differ only in their sialic acid content (Fig. 3a and b), contributing to an increase in identifications in OgpA data. Fig. 3c and d show OgpA cleavage motifs derived from non-specific and semi-tryptic searches, respectively, which match each other and the known cleavage activity N-terminal to *O*-glycosylated threonine and serine residues. As expected, glycan contributions at the P1′ position were dominantly the T-antigen with some Tn-antigen present and negligible amounts of core-2 *O*-glycans.

**Fig. 4:**
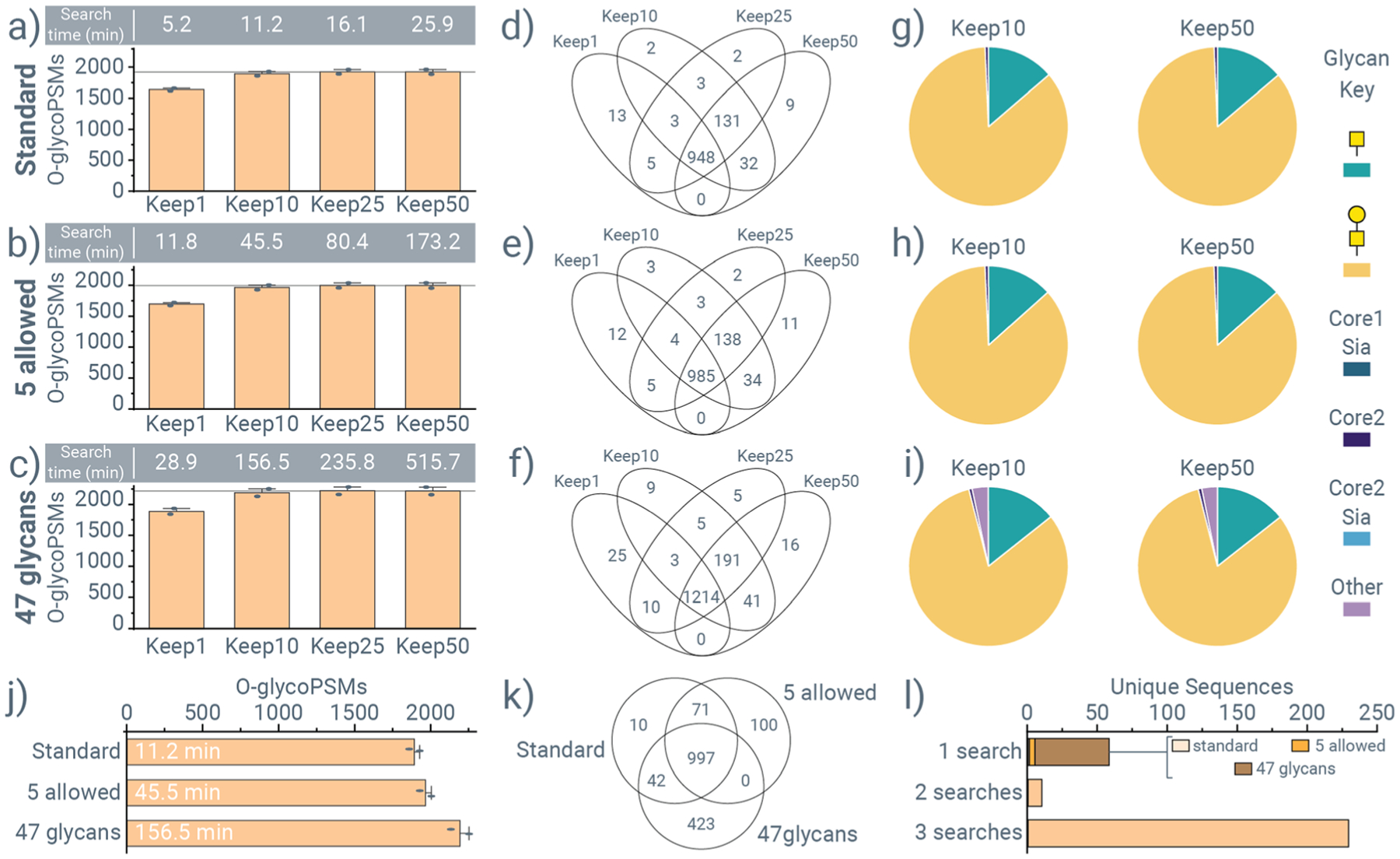
Search times can be lowered by retaining fewer candidate sequences from the open search step. *O*-GlycoPSM identifications for OgpA + trypsin digestions when keeping 1, 20, 25, or 50 (default) candidate sequences for consideration following the open search step in O-Pair Search while conducting (a) a standard semi-tryptic search (4 *O*-glycosites per peptide, 22 *O*-glycan database; “standard”), (b) a semi-tryptic search that allows 5 *O*-glycosites per peptide (“5 allowed”), and (c) a semi-tryptic search that uses a 47 *O*-glycan database (“47glycans”). OgpA digestion here is done concurrently with sialidase treatment according to standard practice. Average search times in minutes are provided above each bar graph, and bars represent the average of two replicates that are also provided as separate data points. The overlap in unique glycopeptide identifications when keeping 1, 20, 25, or 50 candidate sequences is shown for (d) standard, (e) 5 allowed, and (f) 47 glycan searches. Glycan distributions at the P1′ position are shown for Keep10 and Keep50 settings for (g) standard, (h) 5 allowed, and (i) 47 glycans searches. (j) Identifications for the Keep10 setting are shown for the standard, 5 allowed, and 47 glycans searches, with search times in minutes provided. (k) Overlap in unique glycopeptide identifications for standard, 5 allowed, and 47 glycans searches with the Keep10 setting. (l) Unique sequences (amino acid sequence only) that appeared in 1, 2, or 3 searches between standard, 5 allowed, and 47 glycans searches with the Keep10 setting.

**Fig. 5:**
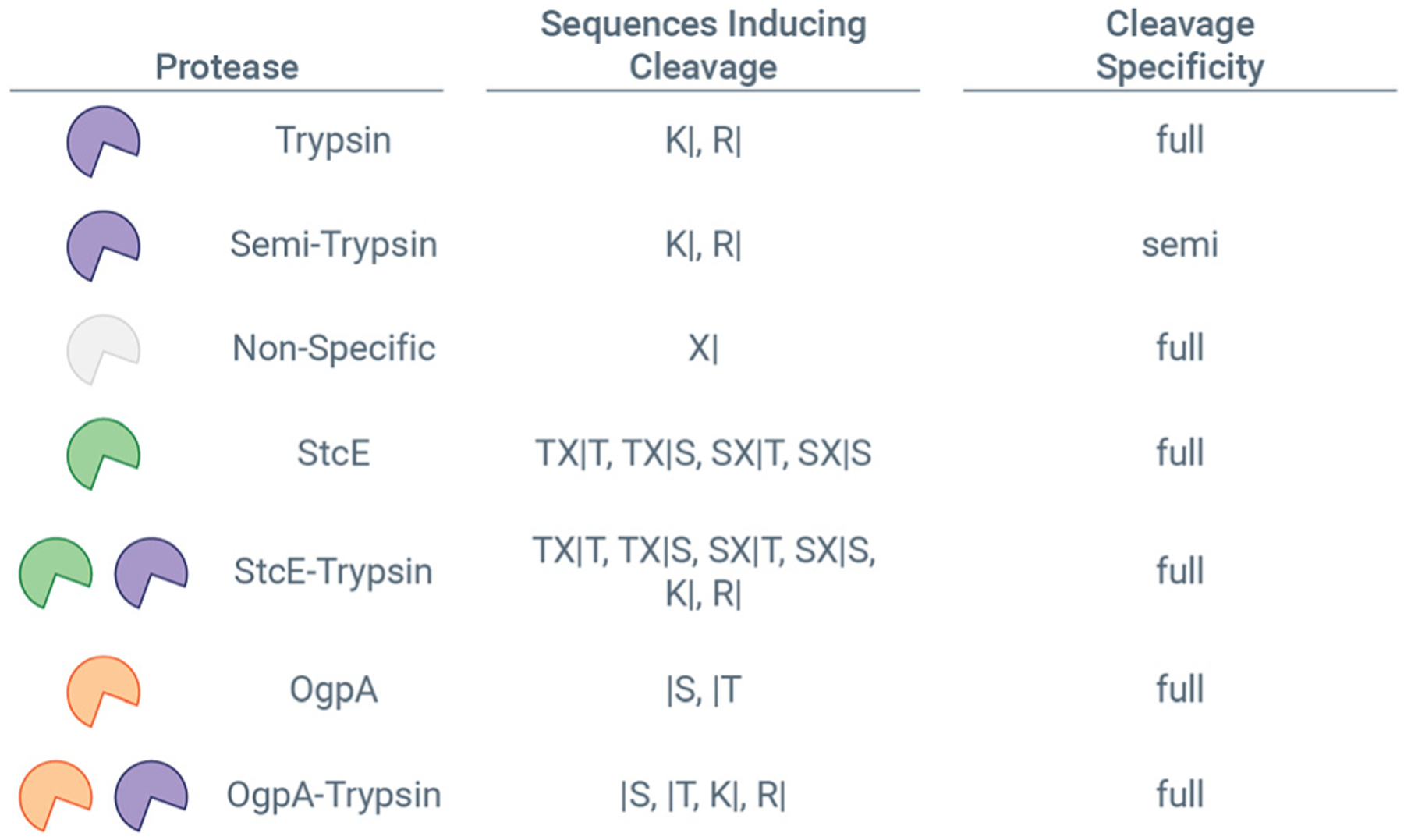
Defining protease cleavage in MetaMorpheus. For each protease, the residues where it cleaves are shown by the single amino acid code (*e.g*., K, R), and the N- or C-terminal cleavage is indicated by the vertical bar (“|”) character. X indicates any amino acid. Cleavage specificity is set to either full or semi to indicate if *in silico* theoretical peptides to consider for identification should follow cleavage rules at both termini (full) or just one (semi). Trypsin, Semi-Trypsin, and Non-Specific are default protease settings in MetaMorpheus. StcE, StcE-Trypsin, OgpA, and OgpA-Trypsin were added user-defined proteases based on data from non-specific and semi-tryptic searches in this study.

In general, search times for non-specific and semi-tryptic searches of StcE and OgpA stayed reasonable (~20–40 minutes) to allow for quick determination of putative cleavage motifs that drastically improves the low-throughput, mostly manual interpretation strategies currently used. The consistent increase in identifications from using the 47-*glycan database*, however, directed us to explore how to decrease search times while still achieving improved *O*-glycoPSM identifications with this expanded glycan database. One key parameter in O-Pair Search is the “*Keep Top N Candidates*” feature that determines how many peptide sequence candidates to consider for *O*-glycopeptide localization following open modification searching. The default setting used in all searches described in Fig. 2 and 3 is to keep 50 candidates. We choose to explore how setting this value to one, ten, and 25 would affect identifications and search times. Fig. 4–c provide results from these different *“KeepN”* parameter values for semi-tryptic searches with (a) standard parameters, (b) the *5allowed* parameter condition, and (c) the 47-*glycan database* parameter condition using the same OgpA dataset from Fig. 3. As expected, the *Keep1* setting is quickest for searches from each of the parameters, but often results in about 15% fewer identifications, negating the benefit of the 47-glycan search. *Keep25* retains effectively the same number of identifications while taking approximately half the search time, but these searches are still approximately 4 hours when using the 47-glycan database. Keeping the top ten candidate sequences (*Keep10*) retains approximately 99% of identifications while requiring only one-fourth to one-third of the search times. Fig. 4d–f show the overlap in *O*-glycopeptide identifications from *Keep1*, *Keep10*, *Keep25*, and *Keep50* settings for the *standard*, *5allowed*, and *47glycan* parameter conditions, respectively, indicating that the vast majority of identifications are shared between the different *Keep* settings. The glycan distributions determined for the cleavage motifs of OgpA are depicted for both *Keep10* and *Keep50* data for the *standard*, *5allowed*, and *47glycans* parameter groups in Fig. 4g–i, respectively. Not only do these data show that *Keep10* and *Keep50* return the same results, but they also underscore the value of the 47-*glycan database* search for providing greater insight into cleavage motifs. Both the *standard* (Fig. 4g) and *5allowed* (Fig. 4h) parameter groups indicate a contribution from T- and Tn-antigen only, but the *47glycan search* (Fig. 4i) shows that OgpA can also tolerate extended core-1 *O*-glycans at the P1′ position. Similar results were obtained for non-specific searches of OgpA data (Fig. S2, ESI†) and non-specific and semi-tryptic searches of StcE (Fig. S3 and S4, ESI†). Finally, we compared identifications from *standard*, *5allowed*, and *47glycan* searches that use the *Keep10* setting in Fig. 4j and show that they share a majority of identified sequences in Fig. 4k. Fig. 4l considers only the underlying sequences of *O*-glycopeptides (without considering the *O*-glycans at specific *O*-glycosites), showing that nearly all sequences are found in all three searches, and those found in only one search come mostly from *47glycan* search. With these benefits in identifications and reasonable search times, we chose to adopt parameters that use the 47-*glycan database* and a *Keep10* setting for all subsequent searches. We note that larger *O*-glycan databases can certainly be explored with O-Pair Search in a similar fashion. A strength of O-Pair Search is that it allows iterations of search spaces like this to enable more exploration of search parameters in *O*-glycoproteomics, which is not feasible with the limitations that exist in canonical search algorithms (discussed above). However, in this experiment with recombinant glycoproteins bearing a limited subset of *O*-glycans, we did not choose to expand our *O*-glycan database further. Our hope is that our community recognizes the value O-Pair Search provides for exploring these search parameters and uses it to characterize biological systems where more complex *O*-glycan databases are relevant.

**Fig. 6:**
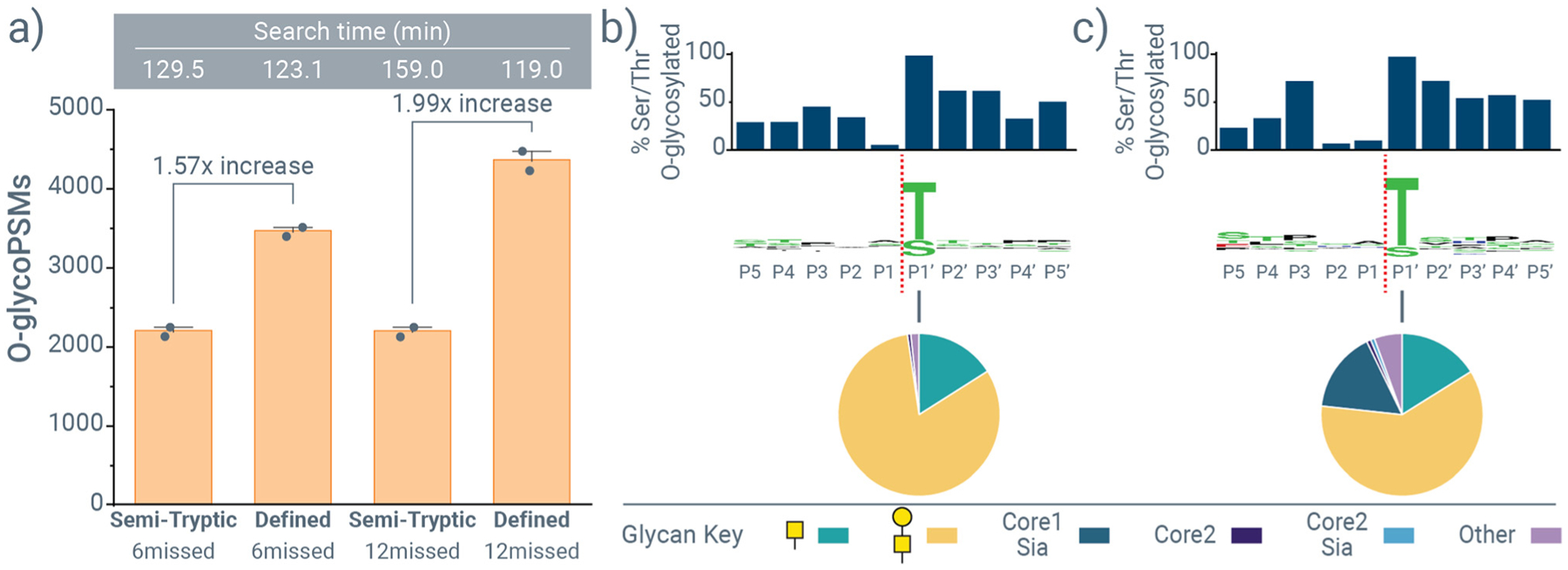
Defining protease specificity settings for OgpA. (a) *O*-glycoPSM identifications for semi-tryptic and defined OgpA-Trypsin searches when allowing 6 or 12 missed cleavages. Search times in minutes are provided above each bar, and bars represent the average of two replicates that are also provided as separate data points. (b) Peptide-glycan cleavage motif for OgpA cleavage (with simultaneous sialidase treatment) generated using data from a defined OgpA-Trypsin search with 12 missed cleavages. (c) Peptide-glycan cleavage motif for OgpA cleavage without a co-incubation of sialidase during OgpA proteolysis generated using data from a defined OgpA-Trypsin search with 12 missed cleavages. Bar graphs above the sequence motifs show the percent of serine and threonine residues observed at a given location that were *O*-glycosylated, and pie graphs show the distribution of glycans observed at P1′.

The cleavage motifs generated from non-specific and semi-tryptic searches provide sufficient insight to generate defined proteolytic settings within the MetaMorpheus environment.^46^ Fig. 5 shows protease cleavages that are present in MetaMorpheus by default, including Trypsin, Semi-Trypsin, and Semi-Tryptic. Based on the data shown in Fig. 2 and 3, StcE and OgpA protease settings can be defined as shown in Fig. 5, and they can be combined with the protease specificities of other proteases, *e.g*., trypsin, that can account for sequential protease treatments like those used in this study. This also means that O-Pair Search can be used to identify *O*-glycopeptides derived from multiple *O*-glycoprotease digestions (*e.g*., cleavage at both N- and C-termini), either through a combination of cleavage motifs in a defined protease setting or through non/semi-specific searches like those used above. We re-searched our StcE and OgpA data using the defined StcE-Trypsin and OgpA-Trypsin settings, respectively. Fig. 6 highlights the benefits using a defined protease settings can have. Based on discussion above about the number of potential *O*-glycosites per peptide, we elected to consider six and twelve missed cleavage events for defined OgpA-Trypsin cleavage, meaning that up to six or twelve serine and threonine residues could be present in theoretical peptide sequence. Semi-tryptic searches with six and twelve missed cleavages returned the same number of identifications (as seen above), while a setting of twelve missed cleavages generated approximately 25% more identifications than a setting of six missed cleavages for the defined search (Fig. 6a). A defined searched with six or twelve missed cleavages returned an approximate 1.5-fold and 2-fold increase in *O*-glycoPSMs, respectively, over a semi-tryptic search (Fig. 6a), highlighting the benefits of defined protease settings where an appropriate search space leads to better score discriminations for false discovery rate calculations. Interestingly, defining the cleavage motif also increased the proportion of identifications from OgpA + trypsin digestion that harbor more than one *O*-glycosite, and the gains in identifications came more from *O*-glycopeptides with two and three *O*-glycosites relative to one *O*-glycosite (Fig. S5, ESI†). Increases were also seen with defined protease setting for StcE data, although benefits were less dramatic (Fig. S6, ESI†). This is likely because of the more complex cleavage motif for StcE relative to OgpA.

With a defined search strategy established, we generated a cleavage motif for OgpA in Fig. 6b using the defined search parameters with 12 missed cleavages (and a 47-glycan database with a Keep10 parameter settings, as described above). This confirms features of the known cleavage motif, including a decidedly non-*O*-glycosylated residue at position P1. We also sought to use our approach to understand how sialylated *O*-glycans might affect the OgpA cleavage motif, so we generated a complementary dataset with sequential OgpA and trypsin digestion on the same *O*-glycoprotein panel, but with the exclusion of the sialidase co-incubation during OgpA digestion. Fig. 6c shows that the peptide sequence motif does not change much, but that some sialylated core-1 *O*-glycans can be tolerated for cleavage by OgpA at P1′. That said, the number of identifications substantially decreased (*vide infra*), matching the reports of lower OgpA efficiency with sialylated *O*-glycans. Overall, these data show how questions surrounding *O*-glycoprotease cleavage preferences can be rapidly explored with our approach even under diverse cleavage conditions, including sialidase co-treatments and *O*-glycoproteases that cleave N- and C-terminally to *O*-glycosites (*e.g*., OgpA and StcE, respectively).

Finally, we sought to use our approach to generate the cleavage motif of immunomodulating metalloprotease (IMPa) from *Pseudomonas aeruginosa*, which has been explored in several recent studies,^35,39,40^ and to evaluate its performance in *O*-glycoproteomic experiments. Like OgpA, IMPa is known to cleave immediately N-terminal to *O*-glycosylated threonine and serine residues, but it does not have the intolerance for sialylated glycans like OgpA. Recent work used synthetic peptides to investigate the importance residues at the P1 position of the substrate peptide for the IMPa’s activity, which showed minimal influence from amino acids adjacent to the cleavage site despite the presence of proline-specific recognition domain that may target the protease to an *O*-glycosylated P–T/S motif.^40^ This work relied on beamCID data only, though, limiting its ability to localize *O*-glycosites for protease motif generation. To add to these studies, we first used non-specific and semi-tryptic searches to generate putative cleavage motifs for IMPa (Fig. S7, ESI†). Similar to OgpA and to previous work on IMPa, our data showed P1′ as an invariably *O*-glycosylated threonine or serine residue, while P1 is a non-*O*-glycosylated residue. Unlike OgpA, IMPa did show some sequence preference for other residues at P1, including alanine and proline residues. Because these features are not dominant (as they are in other *O*-glycoproteases, *e.g*., CpaA^34^), we elected to define the cleavage specificity as |T and |S, analogous to OgpA (Fig. 5).

**Fig. 7:**
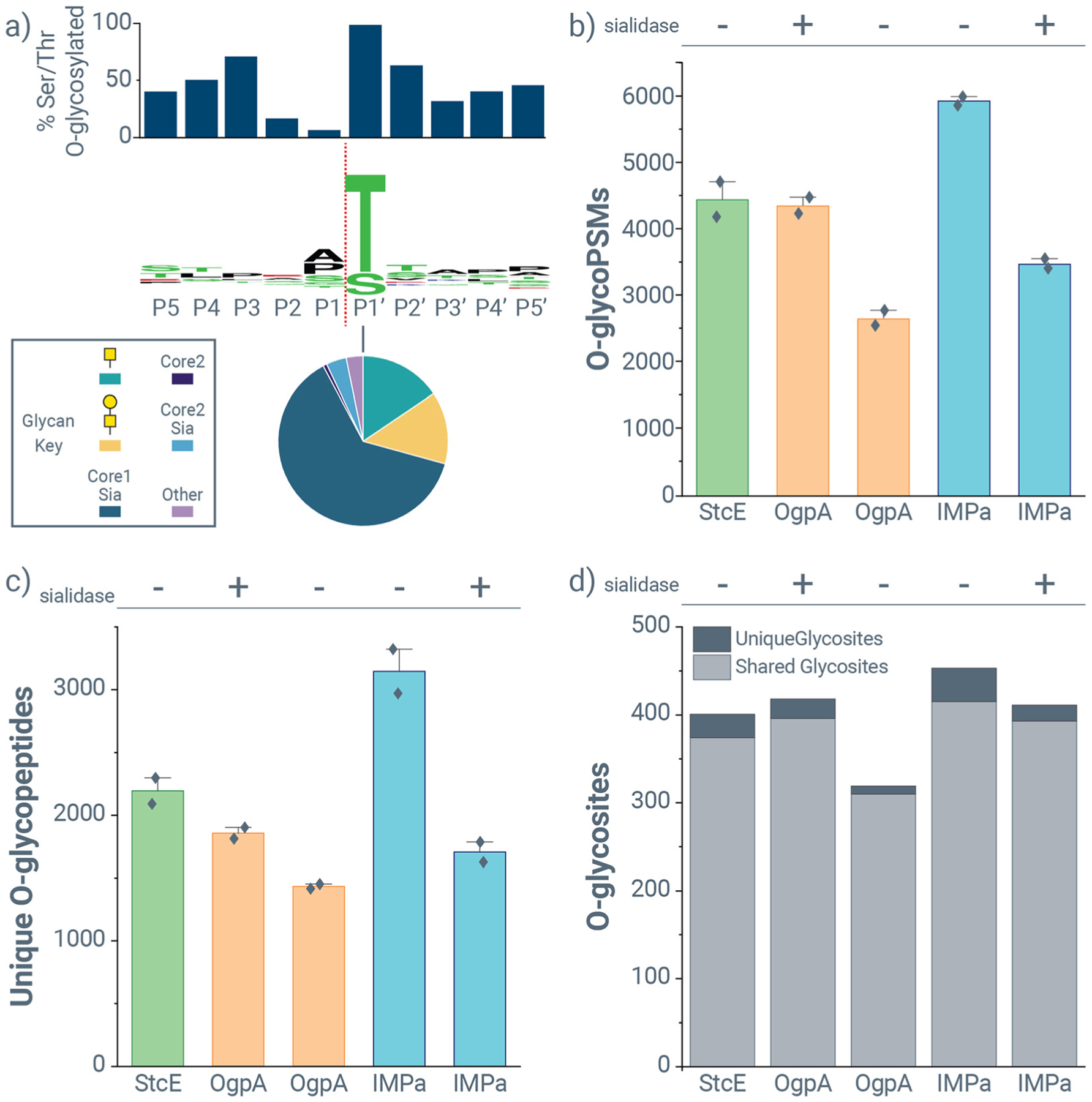
IMPa performance for *O*-glycopeptide identification from mucin-domain glycoproteins. (a) Peptide-glycan cleavage motif for IMPa cleavage generated using data from a defined IMPa-Trypsin search with 12 missed cleavages. Bar graphs above the sequence motifs show the percent of serine and threonine residues observed at a given location that were *O*-glycosylated, and pie graphs show the distribution of glycans observed at P1′. Comparison of (b) *O*-glycoPSM identifications, (c) unique *O*-glycopeptide identifications, and (d) *O*-glycosites for StcE, OgpA, and IMPa digestions with (“+”) and without (“−”) sialidase treatment. In panel d, light gray indicates the number of *O*-glycosites that were detected using other *O*-glycoproteases, and dark gray indicates unique *O*-glycosites only characterized by a given condition. All *O*-glycoprotease treatments included a subsequent trypsin digestion.

Fig. 7a shows the cleavage motif generated when using a defined IMPa-Trypsin search with 12 missed cleavages, a 47-glycan database, with Keep Top N Candidates set to 10. Again, proline and alanine appear at position P1 with slight preference. This is in slight disagreement with work using a peptide library but in more concordance with structural work that indicates a proline-recognition domain and recent unpublished glycoproteomic work.^69^ Even so, our data supports previous work that describes IMPa as a broad specificity *O*-glycoprotease, and the lack of sensitivity to sialylated *O*-glycans is clear based on the *O*-glycan distribution in the pie graph in Fig. 6a. This data also supports that IMPa can cleave at *O*-glycosites with extended core-1 and sialylated and non-sialylated core-2 *O*-glycans. Because of the limitations of the glycan repertoire on recombinant glycoproteins, fucosylated *O*-glycans only comprise ~5% of the total *O*-glycans detected in IMPa experiments. An approximately equivalent fraction (3.6%) of *O*-glycans detected at P1′ with IMPa cleavage have a fucose monosaccharide, showing that fucosylation is tolerated. StcE showed similar prevalence of total *O*-glycan fucosylation (~5%), with *O*-glycans at P2, P1, and P1′ having ~9%, ~2.5%, and ~4% fucosylation. As seen above, OgpA did not tolerate fucosylated *O*-glycans at position P1′ to the same degree (~1% at P1′). These observations will need to be more robustly tested on substrates with more prevalent *O*-glycan fucosylation.

Fig. 7b and c provide context for the number of *O*-glycoPSM and unique *O*-glycopeptide identifications, respectively, for five different digestion conditions that all include a sequential trypsin digestion after *O*-glycoprotease treatment: StcE without sialidase co-incubation (StcE), OgpA with sialidase co-incubation (OgpA), OgpA without sialidase co-incubation (OgpAnoSia), IMPa without sialidase co-incubation (IMPa), and IMPa with sialidase co-incubation (IMPaPlusSia). The decrease in identifications between OgpA and OgpAnoSia underscore the lower efficiency of OgpA for cleaving *O*-glycosites with sialylated *O*-glycans. Similarly, because the decrease in identifications between IMPa and IMPaPlusSia exists for both *O*-glycoPSMs and unique *O*-glycopeptides, these data may indicate that IMPa is more effective at cleaving N-terminal to sialylated rather than non-sialylated *O*-glycans. StcE generally produces more *O*-glycopeptides with 2 or 3 *O*-glycan modifications, which matches its cleavage proclivity toward sequences where there may be two adjacent *O*-glycosites (Fig. S8, ESI†). Also, *O*-glycopeptides derived from OgpA and IMPa digestion tend to have more than one *O*-glycosite for about 75% of identifications, indicating they often have missed cleavages (*i.e*., internal *O*-glycosites). Search strategies for experiments employing these *O*-glycoproteases must account for internal *O*-glycosites present in the peptide sequence, including the use of electron-based fragmentation to complement collisional dissociation to minimize false N-terminal glycan composition determination.^58^

StcE, OgpA, and IMPa identify *O*-glycopeptides covering largely the same *O*-glycosites (Fig. 7d), even though IMPa identifies the most unique and shared *O*-glycosites. IMPaPlusSia identifies as many *O*-glycosites as StcE or OgpA, and surprisingly, OgpA identifies more *O*-glycosites than StcE. That said, OgpA practically requires de-sialylation, which inherently collapses the heterogeneity of *O*-glycans and limits the number of *O*-glycoforms that can be characterized. Thus, StcE has more utility than OgpA for *O*-glycoproteomics of densely *O*-glycosylated mucin-domain glycoproteins. IMPa clearly outperforms OgpA, making it widely useful in a number of *O*-glycoproteomic applications, and it also outperforms StcE for mucin-domain glycoprotein characterization. Overall, these data further elucidate the impact of peptide substrate sequence on IMPa activity and show the utility of a broad specificity *O*-glycoprotease (without sialylation sensitivity) for *O*-glycoproteomic applications.

## Conclusions

This study explores parameters that affect identifications and search times when using O-Pair Search for *O*-glycoproteomics and provides a template for using O-Pair Search results to rapidly map *O*-glycoprotease substrate preferences. Search times for O-Pair Search remain within reasonable time frames even when considering multiple *O*-glycosites per peptide and using modest sized *O*-glycan databases with 20–50 glycan compositions. The speed of search strategies like those used by O-Pair Search are key for future *O*-glycoproteomics work, both in general and for *O*-glycoprotease motif mapping. O-Pair Search enables straightforward exploration of multiple parameters that affect data quality and *O*-glycoPSM identifications, which can take prohibitively long when searching *O*-glycopeptide data with other platforms. Importantly, O-Pair Search is freely available and open-source, with easy installation and operation instructions available at: https://github.com/smith-chem-wisc/MetaMorpheus.

Beyond providing a platform to interrogate *O*-glycoprotease substrate preferences, our data underscores several important features of *O*-glycoproteomics analysis, including the notion that consideration of three to five *O*-glycosites per peptide should be sufficient for most applications using *O*-glycoproteases for digestion. This work also demonstrates the utility of IMPa as a broadly activity *O*-glycoprotease that can be useful of densely *O*-glycosylated mucin-domain *O*-glycoproteins, and it adds to the data describing the subtle sequence preferences of proline and alanine at P1 for IMPa cleavage. We note that this approach can also be used to map proteolytic cleavage preferences for any protease, including those like Cathepsin D that digest highly glycosylated *O*-glycoproteins like mucins but are not professional *O*-glycoproteases.^70^ Finally, we recognize there is currently no search algorithm that allows cleavage to be defined by the presence of specific post-translational modification at a specific residue. Addition of this feature to glycoproteomics search algorithms would greatly improve both *O*-glycoprotease cleavage motif investigations that seek to better understand their biological functions and also the growing number *O*-glycoproteomic studies that rely on this emerging class of proteases to generate MS/MS-amenable *O*-glycopeptides.

## Supplementary Material

Supplemental info 1

Supplemental info 2

Supplemental info 3

## Data Availability

The mass spectrometry raw data, FASTA sequence database, and search results have been deposited to the ProteomeXchange Consortium *via* the PRIDE partner repository with the dataset identifier PXD035775.^56^
