# Supplementary material for: Deciphering O-glycoprotease substrate preferences with O-Pair Search†
Source: Mol Omics. Author manuscript; Available in PMC 2023 Mar 13. (PMC10010678; doi:10.1039/d2mo00244b)
Supplement: Supplemental info 1 [file NIHMS1878830-supplement-Supplemental_info_1.pdf]

Electronic Supplementary Material (ESI) for Molecular Omics.
This journal is © The Royal Society of Chemistry 2022

Please use files tab to view ESI tables.
