# Supplementary material for: Deciphering O-glycoprotease substrate preferences with O-Pair Search†
Source: Mol Omics. Author manuscript; Available in PMC 2023 Mar 13. (PMC10010678; doi:10.1039/d2mo00244b)
Supplement: Supplemental info 2 [file NIHMS1878830-supplement-Supplemental_info_2.pdf]

Electronic Supplementary Material (ESI) for Molecular Omics.
This journal is © The Royal Society of Chemistry 2022

Deciphering O-glycoprotease substrate preferences with O-Pair Search

Nicholas M. Riley,[1] Carolyn R. Bertozzi[1,2]

[1]Department of Chemistry, Sarafan ChEM-H, Stanford University, Stanford, California, USA
[2]Howard Hughes Medical Institute, Stanford, California, USA

Supplemental Figures and Tables

Supplemental Figure 1. Comparison of Byonic results with O-Pair Search.
Supplemental Figure 2. Glycan distributions a the P1' position for OgpA proteolysis with a non-specific search and a Keep10 setting.
Supplemental Figure 3. Glycan distributions a the P2, P1, and P1' positions for StcE proteolysis with a non-specific search and a Keep10 setting.
Supplemental Figure 4. Glycan distributions a the P2, P1, and P1' positions for StcE proteolysis with a semi-tryptic search and a Keep10 setting.
Supplemental Figure 5. Distribution of O-glycosites per peptide for OgpA+trypsin digestions with various search settings
Supplemental Figure 6. Defining protease specificity for StcE.
Supplemental Figure 7. IMPa peptide-glycan cleavage motifs.
Supplemental Figure 8. Distribution of O-glycosites per peptide for various O-glycoprotease digestions.

Supplemental Table 1. Description of search parameters that can be defined by the user in an O-Pair Search within MetaMorpheus.
Supplemental Table 2. Description of the various search settings and their short name condition names for searches used throughout this study.

Supplemental Data (separate file)

Supplemental Data 1. Database of 22 O-glycan compositions.
Supplemental Data 2. Database of 12 O-glycan compositions.
Supplemental Data 3. Database of 47 O-glycan compositions.

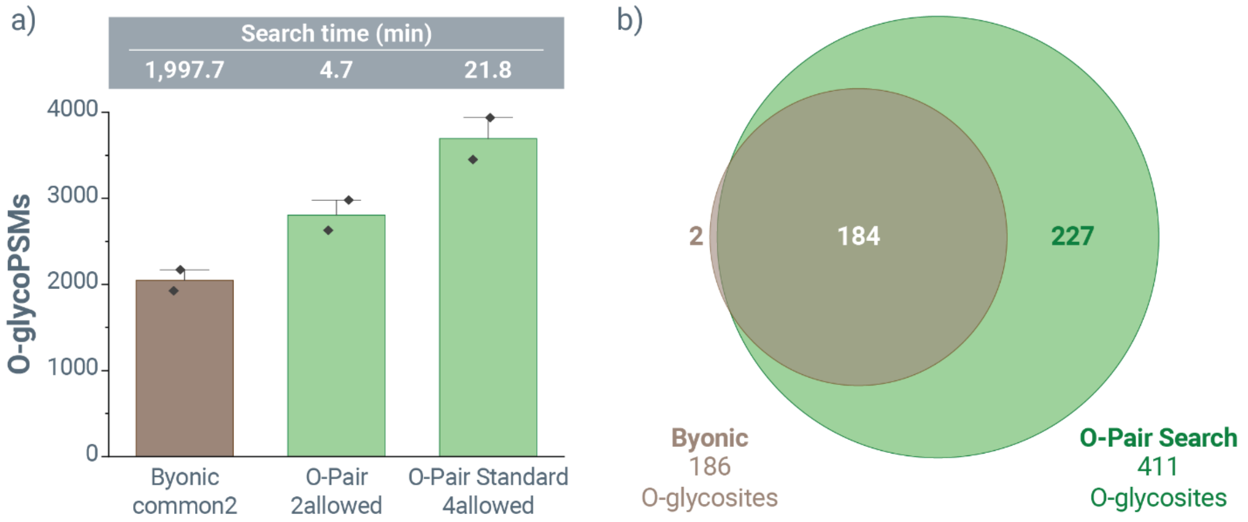

Supplemental Figure 1. Comparison of Byonic results with O-Pair Search. **a)** O-glycoPSMs (bars) and search times (top) are shown for three different semi-tryptic searches of the StcE-trypsin data, including Byonic with O-glycans set as "common2", O-Pair Search with the "2allowed" setting, and O-Pair Search with standard settings. Details on O-Pair Search settings are available in Supplemental Table 2. For all three searches, 3 missed cleavages with semi-tryptic specificity were allowed, and a 22-glycan database was used. Average search times in minutes are provided above each condition, and bars represent the average of two replicates that are also provided as separate data points. **b)** The overlap in O-glycosites detected between the Byonic "common2" search and the "2allowed" O-Pair Search.

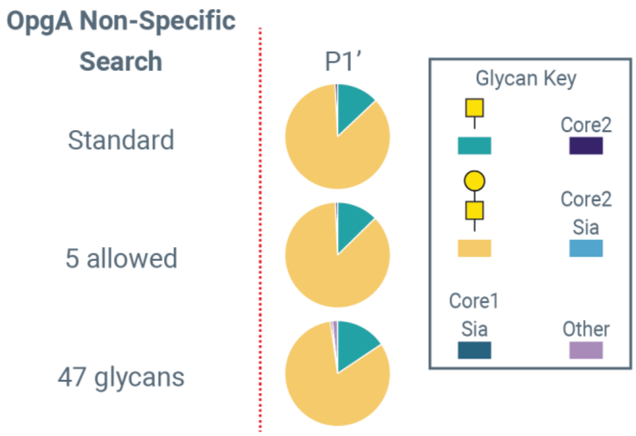

Supplemental Figure 2. Glycan distributions a the P1' position for OgpA proteolysis with a non-specific search and a Keep10 setting. Distributions are for a standard non-specific search (4 O-glycosites per peptide, 22 O-glycan database; "standard"), a non-specific search that allows 5 O-glycosites per peptide ("5 allowed"), and a non-specific search that uses a 47 O-glycan database ("47glycans").

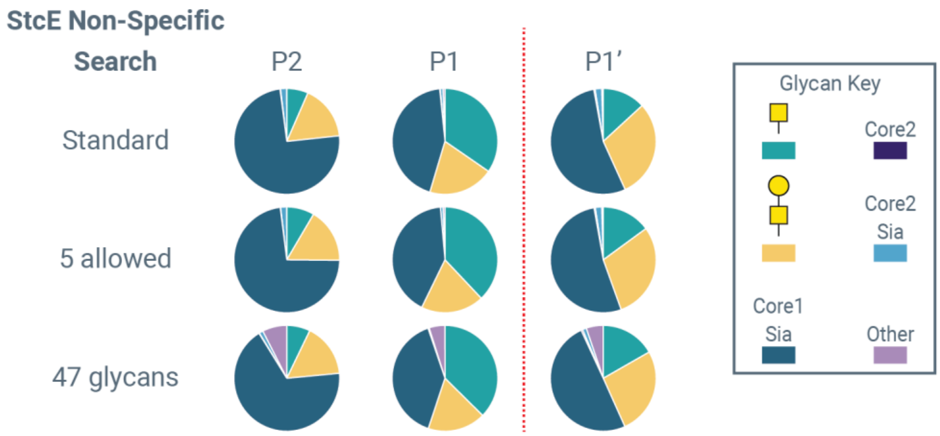

Supplemental Figure 3. Glycan distributions a the P2, P1, and P1’ positions for StcE proteolysis with a non-specific search and a Keep10 setting. Distributions are for a standard non-specific search (4 O-glycosites per peptide, 22 O-glycan database; “standard”), a non-specific search that allows 5 O-glycosites per peptide (“5 allowed”), and a non-specific search that uses a 47 O-glycan database (“47glycans”).

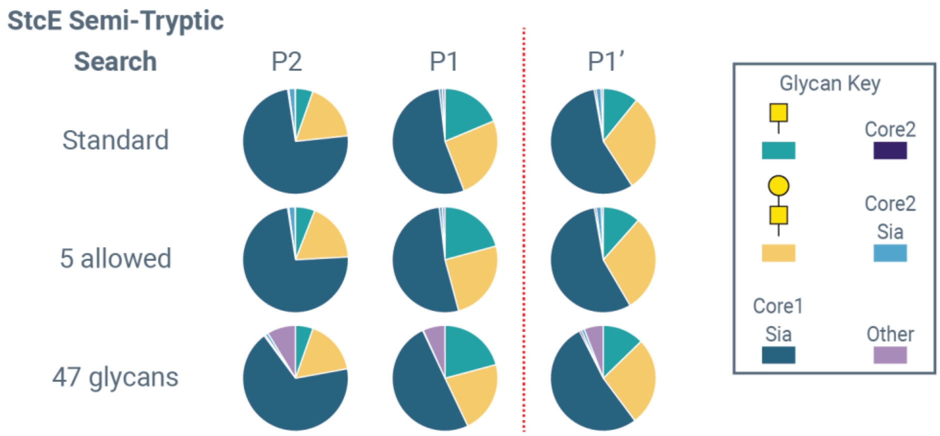

Supplemental Figure 4. Glycan distributions a the P2, P1, and P1’ positions for StcE proteolysis with a semi-tryptic search and a Keep10 setting. Distributions are for a standard semi-tryptic search (4 O-glycosites per peptide, 22 O-glycan database; “standard”), a semi-tryptic search that allows 5 O-glycosites per peptide (“5 allowed”), and a semi-tryptic search that uses a 47 O-glycan database (“47glycans”).

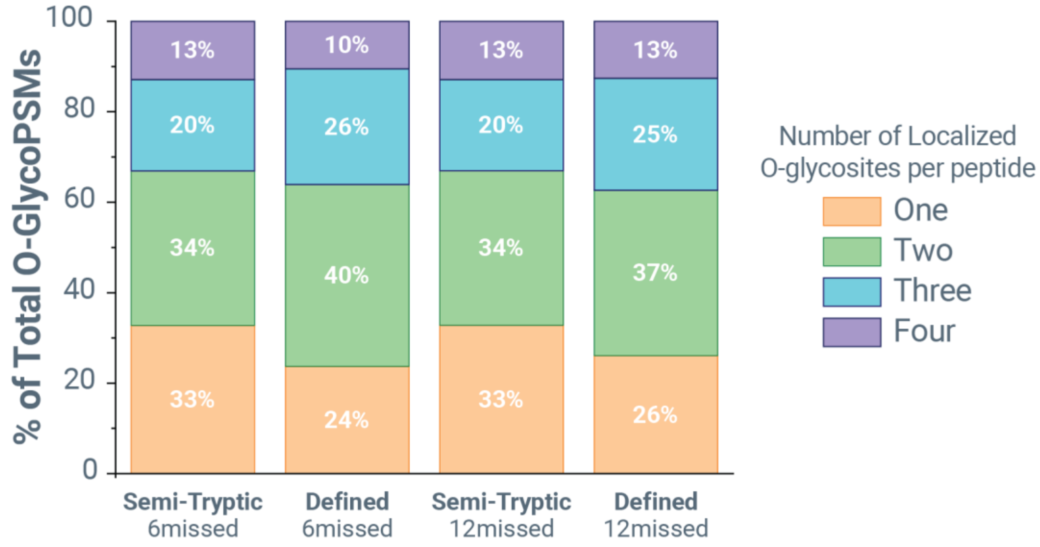

Supplemental Figure 5. Distribution of O-glycosites per peptide for OgpA+trypsin digestions with various search settings. Stacked bar graphs show the proportion of O-glycoPSM identifications that had one, two, three, or four modified O-glycosites in O-glycoPSM identifications for semi-tryptic and defined OgpA-Trypsin searches when allowing 6 or 12 missed cleavages.

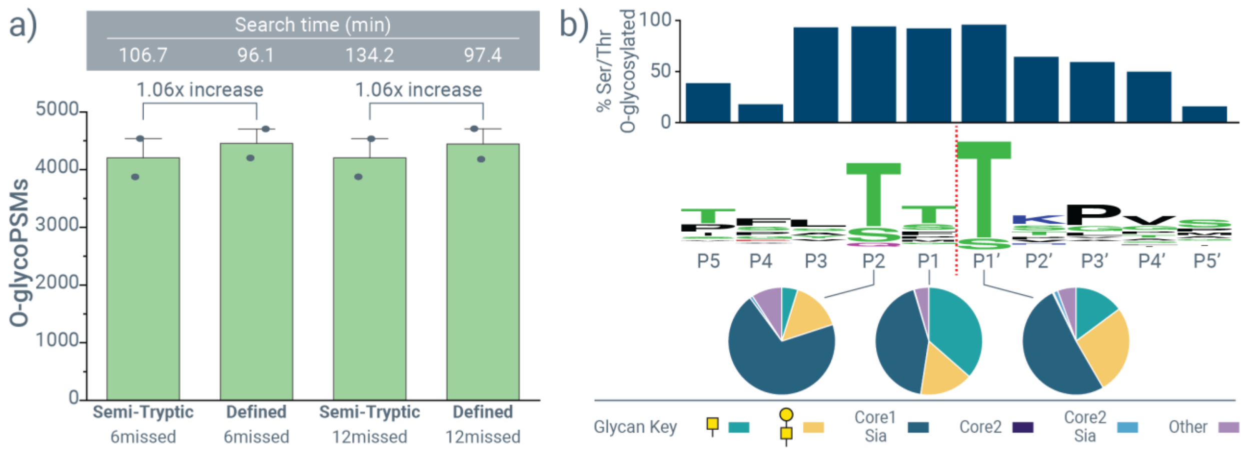

Supplemental Figure 6. Defining protease specificity for StcE. a) O-glycoPSM identifications for semi-tryptic and defined StcE-Trypsin searches when allowing 6 or 12 missed cleavages. Bars represent the average of two replicates that are also provided as separate data points. **b)** Peptide-glycan cleavage motif for OgpA cleavage generated using data from a defined OgpA-Trypsin search with 12 missed cleavages. Bar graphs above the sequence motifs show the percent of serine and threonine residues observed at a given location that were O-glycosylated, and pie graphs show the distribution of glycans observed at P2, P1, and P1’.

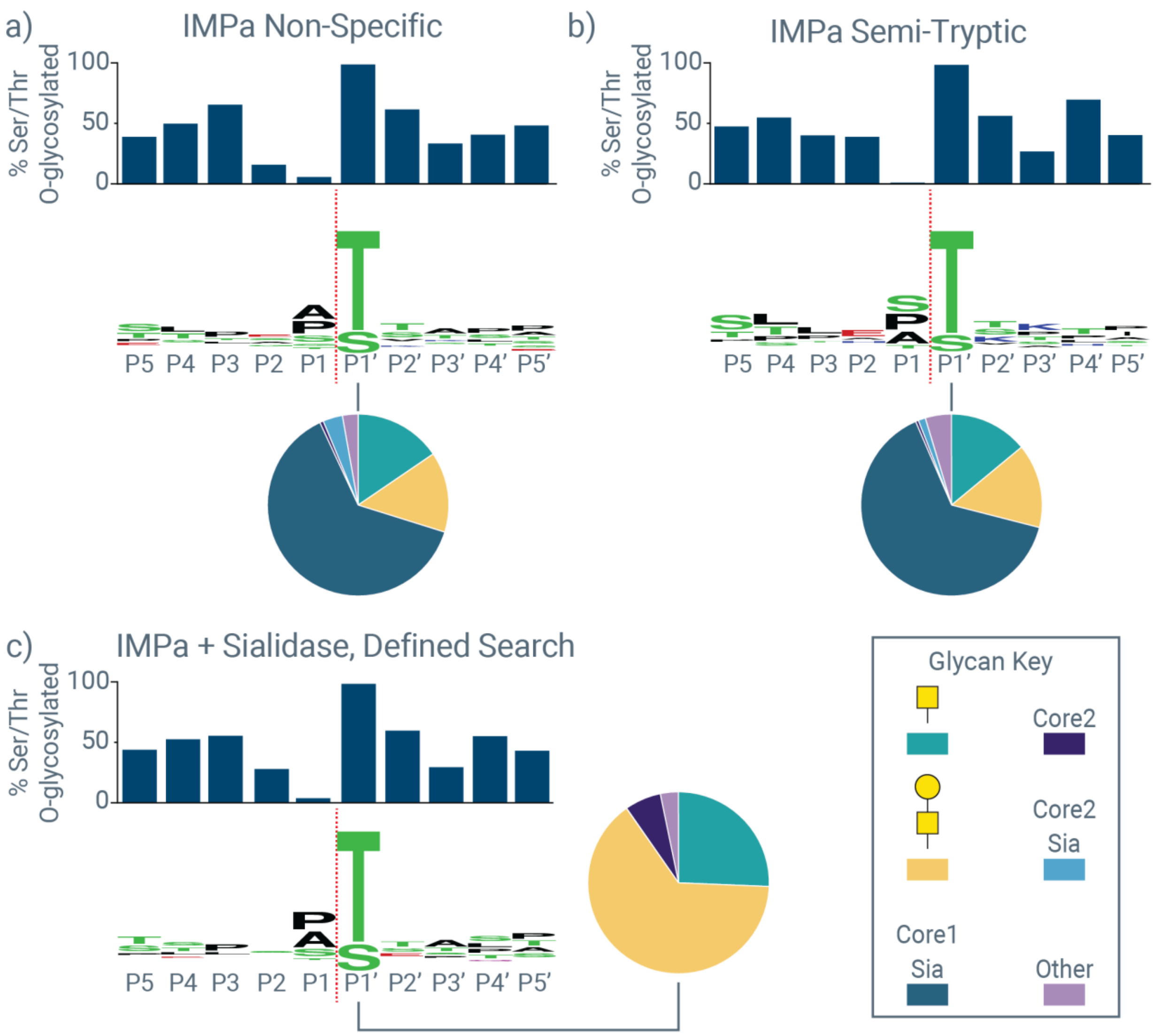

Supplemental Figure 7. IMPa peptide-glycan cleavage motifs. Peptide-glycan cleavage are shown for IMPa for a) a non-specific search, b) a semi-tryptic search, and c) a defined search where IMPa digestion included co-incubation with sialidase. All three searches use a 47 glycan database and a Keep10 setting. Bar graphs above the sequence motifs show the percent of serine and threonine residues observed at a given location that were O-glycosylated, and pie graphs show the distribution of glycans observed at P1'.

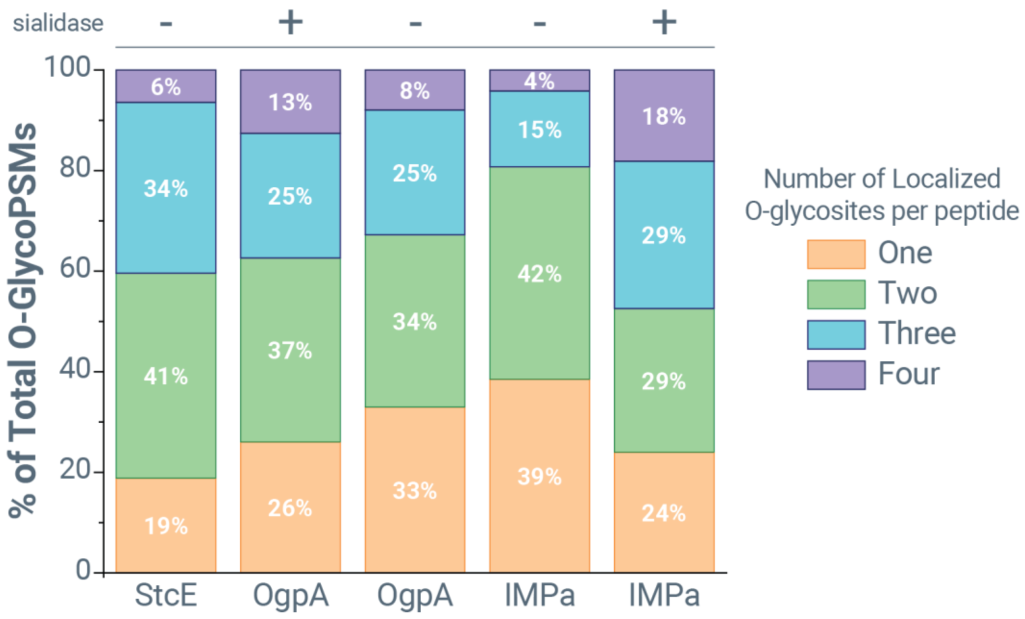

Supplemental Figure 8. Distribution of O-glycosites per peptide for various O-glycoprotease digestions. Stacked bar graphs show the proportion of O-glycoPSM identifications that had one, two, three, or four modified O-glycosites in StcE, OgpA, and IMPa digestions with ("+") and without ("-") sialidase treatment. All O-glycoprotease treatments included a subsequent trypsin digestion.

Parameter	Description	Setting in Standard Search	Static Through All Experiments
Glycan Database	User-provided glycan compositions to use for glycopeptide identification	22 glycan database	No
Keep top N Candidates	Number of candidate peptide sequences to consider following open modification searching	50	No
Dissociation Type	Fragmentation method used for MS/MS scans	HCD	Yes
Child Scan Type	Fragmentation method used for MS/MS scans if paired spectra were generated using product-dependent triggering	EThcD	Yes
Maximum Oglycan Allowed	Maximum number of Oglycan modifications permitted on a single peptide sequence	4	No
OxoniumIonFilt	A filter that requires the presence of oxonium ions to consider spectra for glycopeptide identification	Yes (checked)	No
Use Provided Precursors	Use the precursor information provided by the scan header of the raw data	Yes (checked)	No
Deconvolute Precursors	Deconvolute precursor m/z values into mass values	Yes (checked)	No
Trim MS1 Peaks	Remove peaks in MS1 scans below a certain rank or ratio to base peak intensity	No (unchecked)	No
Trim MS2 Peaks	Remove peaks in MS2 scans below a certain rank or ratio to base peak intensity	No (unchecked)	No
Generate Decoy Proteins	Appended decoy proteins to the user-provided protein sequence database, either as reversed sequences or slided decoys (choose one or the other option)	Yes (checked), reversed decoys	No
Protease	Which cleavage motif to use to generate in silico theoretical peptides to consider	non-specific or semi-trypsin	No

Supplemental Table 1. Description of search parameters that can be defined by the user in an O-Pair Search within MetaMorpheus. The list is not exhaustive but includes all parameters tested in this study. The table is continued on the next page.

Parameter	Description	Setting in Standard Search	Static Through All Experiments
Max Missed Cleavages	The maximum number of missed cleavages allowed for a selected protease	59 or 3	No
Initiator Methionine	Whether or not to remove initiator methionine residues from user-provided protein sequences	Variable	Yes
Max Modification Isoforms	Maximum number of possibilities to consider for non-glycan modifications	1024	Yes
Max Modification Per Peptide	Maximum number of variable non-glycan modifications to consider	2	Yes
Min Peptide Len	Mimimum residue length for a peptide to be identified	5	Yes
Max Peptide Len	The maximum residue length to consider for peptide sequences	60	No
Precursor Mass Tolerance	Mass error tolerances allowed for precursor mass measurements from MS1 scans	10 ppm	Yes
Product Mass Tolerance	Mass error tolerances allowed for product ion mass measurements from MS2 scans	20 ppm	Yes
Minimum score allowed	Lower threshold for Morpheus score required to retain an identification	3	Yes
Max Threads	Number of cores to use in the search	16	Yes
Fixed Modifications	Modifications to always include on specific amino acids	Carbaminothylation on Cysteine	Yes
Variable Modifications	Potential modifications to consider for specific amino acids	Oxidation on Methionine, Deamidation on Asparagine	No

Supplemental Table 1 continued. Description of search parameters that can be defined by the user in an O-Pair Search within MetaMorpheus. The list is not exhaustive but includes all parameters tested in this study.

Condition	Setting
25aa	Max Peptide Len set to 25; only for Non-Specific searches
60aa*	Max Peptide Len set to 60; used in Non-Specific searches; *indicates this is the standard search for the Non-Specific condition; all "Standard" Non-Specific results are from this
3miss*	Max Missed Cleavages set to 3; used in Semi-Tryptic searches; *indicates this is the standard search for the Semi-Tryptic condition; all "Standard" Semi-Tryptic results are from this
6miss	Max Missed Cleavages set to 6; used in Semi-Tryptic and Defined searches
9miss	Max Missed Cleavages set to 9; used in Semi-Tryptic searches
12miss	Max Missed Cleavages set to 12; used in Semi-Tryptic and Defined searches
2allowed	Maximum Oglycan Allowed set to 2
3allowed	Maximum Oglycan Allowed set to 3
5allowed	Maximum Oglycan Allowed set to 5
12glycans	Use a database of 12 O-glycan compositions
47glycans	Use a database of 47 O-glycan compositions
Slided	Use slided decoy setting instead of reversed
NoDeamid	Deamidation of asparagine not included as variable
Indiv	Each raw file searched individually and then concatenated rather than processed in-batch within the same search
MinScore5	Minimum score allowed set to 5 instead of 3
MinScore10	Minimum score allowed set to 10 instead of 3
TrimMS1	Peaks in MS1 spectra were trimmed to the top 1000 and 0.01 of base peak ratio
TrimMS2	Peaks in MS2 spectra were trimmed to the top 1000 and 0.01 of base peak ratio
NoOxFilter	OxoniumIonFilt was unchecked, removing requirement for oxonium ions to be considered for glycopeptide ID
Keep1	Keep top N Candidates was set to 1
Keep10	Keep top N Candidates was set to 10
Keep25	Keep top N Candidates was set to 25
Defined	A defined cleavage motif was created and used rather than non-specific or semi-tryptic

Supplemental Table 2. Description of the various search settings and their short name condition names for searches used throughout this study.
